# A comparative analysis of solitary suicides, suicides following homicide, and suicide pacts using the National Violent Death Reporting System

**DOI:** 10.1186/s12888-022-04495-w

**Published:** 2023-01-03

**Authors:** Jenna Ashley, Kawon Victoria Kim, Cayley Russell, Shannon Lange

**Affiliations:** 1 Health Research Methods, Evidence and Impact, McMaster University, 1280 Main St. W, L8S 4K1 Hamilton, ON Canada; 2 Institute for Mental Health Policy Research, Centre for Addiction and Mental Health, 33 Ursula Franklin St, M5S 2S1 Toronto, ON Canada; 3 Campbell Family Mental Health Research Institute, Centre for Addiction and Mental Health, 250 College Street, M5T 1R8 Toronto, ON Canada; 4 Department of Psychiatry, University of Toronto, 250 College St. 8th Floor, M5T 1R8 Toronto, ON Canada

**Keywords:** Suicide, Suicide following homicide, Suicide pact, NVDRS, United States

## Abstract

**Background:**

Incidents of suicide can be categorized into three main types: solitary suicides, suicides following homicide, and suicide pacts. Although these three suicide incidents vary by definition, no studies to-date have simultaneously examined and compared them for potential differences. The objective of the current study was to empirically and descriptively compare solitary suicides, suicides following homicide, and suicide pacts in the United States.

**Methods:**

Restricted-access data from the National Violent Death Report System for 2003–2019 for 262,679 solitary suicides, 4,352 suicides following homicide, and 450 suicide pacts were used. Pairwise comparisons of the three suicide incident types were made for demographic factors, method of suicide, preceding circumstances, mental health status, and toxicology findings.

**Results:**

Solitary suicides, suicides following homicide, and suicide pacts have distinct profiles, with statistically significant (*p* < 0.05) differences across all pairwise comparisons of sex, race, ethnicity, marital status, education, method of suicide, financial problems, interpersonal relationship problems, physical health problems, mental health problems, mood disorders, suicide attempt history, and opiate use at the time of death.

**Conclusion:**

Despite sharing a few commonalities, solitary suicides, suicides following homicide, and suicide pacts represent distinct phenomena. Each of these suicide incident types likely have their own unique prevention pathways.

**Supplementary Information:**

The online version contains supplementary material available at 10.1186/s12888-022-04495-w.

## Background

In 2020 in the United States (US), suicide was among the ten leading causes of death among individuals 10–64 years of age [1]. About 46,000 people died by suicide in the US in 2020, for an age-standardized suicide mortality rate of 13.5 per 100,000 population [1]. The fact that these numbers include decedents of different types of suicide incidents is rarely acknowledged. In addition to decedents of solitary suicides (a suicide that involves only one decedent), suicide statistics include decedents who died by suicide following homicide, for which the suicide decedent was the perpetrator, as well as decedents who died by suicide together as part of a pact. Despite suicides following homicide and suicide pacts being relatively rare in comparison to solitary suicides (approximately 2% and less than 1% of all suicides, respectively [2–4]), the differentiation of such incidents may have important prevention implications.

It has been found that compared to decedents of solitary suicides, decedents of suicides following homicide are more likely to have been male, non-white, and perpetrators of domestic violence [4–7]. With respect to comparing suicide pacts with solitary suicides, the literature is sparse and outdated, with only two existing studies, both of which use nationwide samples from England and Wales [2, 8]. Based on these studies, compared to decedents of solitary suicides, decedents of suicide pacts are more likely to have been middle-aged, in a relationship or married, and suffering from a physical illness [2, 8]. Although there are studies that compare suicides following homicide and suicide pacts to solitary suicides individually, no study to date has compared these two types of suicide incidents to one another.

Given the relative rarity of suicides following homicide and suicide pacts, existing studies are often plagued by small sample sizes. Further, existing studies on such incidents often rely on data sources with limited information (e.g., death certificates). As a result, the characteristics used to describe decedents of suicide following homicide and suicide pacts have been extremely limited in scope. For instance, there is a dearth of research comparing the preceding circumstances, mental health status, and toxicology findings of decedents of solitary suicides, suicides following homicides, and suicide pacts. This represents a significant gap in the literature, as an understanding of such characteristics and the differences between the three types of suicide incidents may provide insight into points of contact where intervention and prevention can occur. Thus, the objective of the current descriptive study was to empirically compare the demographic factors, method of suicide, preceding circumstances, mental health status, and toxicology findings between decedents of solitary suicides, suicides following homicides, and suicide pacts.

## Materials and methods

### Data source

The current study used restricted-access data from the National Violent Death Reporting System (NVDRS) – a state-based active surveillance system in the US that provides a detailed account of violent deaths occurring in participating states–for 2003 to 2019. The NVDRS provides de-identified, multistate, incident-level data, comprised of hundreds of unique variables derived from numerous sources including law enforcement records, coroner and medical examiner reports, death certificates, and toxicology reports [9]. Only a small number of states participated in 2003 (seven), however, by 2019, data were available for a total of 44 states (see Table A1 in the Appendix). For more information on the NVDRS, please see [9].

### Measures

For the purpose of the current study, the following three distinct categories of suicide incidents were derived from the NVDRS: solitary suicides, suicides following homicide, and suicide pacts. A solitary suicide was defined as a suicide involving only one decedent. Solitary suicides were ascertained using the incident category variable in the NVDRS by selecting “single suicide”. Consistent with the current literature, a suicide pact was defined as “a mutual arrangement between [at least] two people who resolve to die at the same time and, nearly always, in the same place” [3]. Suicide pact decedents were identified by first using the incident category variable in the NVDRS by selecting “multiple suicide”. Then, as per the recommendation of the NVDRS, for incidents categorized as “multiple death – other”, the coroner/medical examiner and law enforcement narratives were reviewed and coded as a suicide pact if they contained explicit statements, clear implications, or strong evidence to infer that there was an agreement between the two decedents to die by suicide. If the manner of death was undetermined for one suicide pact member or one member of the pact died by assisted suicide, the respective incident was not included. Incidents that involved collateral deaths of children were also excluded. The incident identification number (a unique identifier for each incident, also used to link victims) was used to identify the members of each suicide pact. All potential incidents of a suicide pact were discussed during case conferences, and were only retained as a suicide pact if consensus was reached. Suicides following homicide were defined as one person killing one or more others and then dying by suicide within 24 h. It should be noted that the current study only included data on the perpetrator of the homicide, who is the suicide decedent.

Demographic factors included age, education, marital status, military history, race/ethnicity, and sex. Race (Asian/Pacific Islander/American Indian/Alaska Native, Black or African American, and White) and ethnicity (Hispanic, or Non-Hispanic) were compared separately; however, due to low cell counts, the p-values are provided but the proportions are not presented in any figures or tables in order to comply with the NVDRS data sharing agreement. Preceding circumstances, which were suspected to have contributed to the decedent’s death, included the death or suicide of a family member or friend, financial problem, homelessness, home loss or eviction, interpersonal relationship problems, job or school problems, legal problems, and physical health problems. The NVDRS defines a “crisis” as any of the former, as well as mental health or substance use crises, if they occurred within 14 days of their death; if endorsed, the respective problem or event was thought to have contributed to the death of the suicide decedent. Mental health status variables of interest included mental health problems (i.e., any mental health problem in the Diagnostic and Statistical Manual of Mental Disorders, 5th Revision (DSM-5) with the exception of substance abuse disorders that the victim was experiencing at the time of their death; defined by the NVDRS [9]), mood disorder diagnosis (bipolar disorder or depression/dysthymia), suicide attempt history, and suicide intent disclosure (i.e., whether the victim disclosed suicidal thoughts to anyone in the month preceding their death). Method of suicide was categorized as active or passive. Passive methods included intentional self-poisonings (ICD-10 codes: X60-X69), whereas active methods included all other methods of suicide (ICD-10 codes: X70-X83). Suicide incidents that were coded as *intentional self-harm by unspecified means* (ICD-10 code: X84) were excluded from the method of suicide analyses. If an incident did not have a suicide-specific ICD-10 code as the method of death, the coroner/medical examiner and law enforcement narratives, as well as information available for the variables cause of death and weapon type, were reviewed to ascertain the method of suicide. Those without enough information to determine if the method was active or passive were excluded from the respective analyses. Toxicology findings of interest included blood alcohol concentration (BAC), as well as presence of amphetamines, cocaine, and opiates; only individuals who underwent a toxicological examination were included in the analyses on the respective measures. BAC, a continuous measure, was categorized as ≥ 0.08 g/dl or < 0.08 g/dl (the legal limit for impaired driving in the US). As per the recommendations of the NVDRS, cases where the BAC was > 0.60 g/dl were excluded as they are suspected to be in error, given that a BAC above this threshold is highly unlikely [10].

### Statistical analysis

The only continuous variable, age, was analyzed using a one-way analysis of variance. If the analysis of variance showed that there was a statistically significant difference between the means of at least two of the groups, the Tukey test was conducted for pairwise comparisons. For categorical variables, Pearson’s Chi-Square test was used; when statistically significant, the three groups were then tested pairwise with Pearson’s Chi-Square, using the Bonferroni method to adjust the p-value. Mean difference for continuous variables and odds ratios (ORs) for categorical variables, with 95% confidence intervals (CI), were calculated for all statistically significant pairwise comparisons. ORs were estimated using conditional maximum likelihood estimation. If decedents were missing information for a variable, they were excluded from the total count (n) when calculating proportion and running statistical tests for that variable. All analyses were conducted using R version 4.0.5 [11]. Statistical significance was determined using an α of 0.05.

## Results

In total, there were 262,679 decedents of solitary suicides, 4,352 decedents of suicides following homicide, and 450 decedents of suicide pacts.

### Demographics and method of suicide

**Fig. 1:**
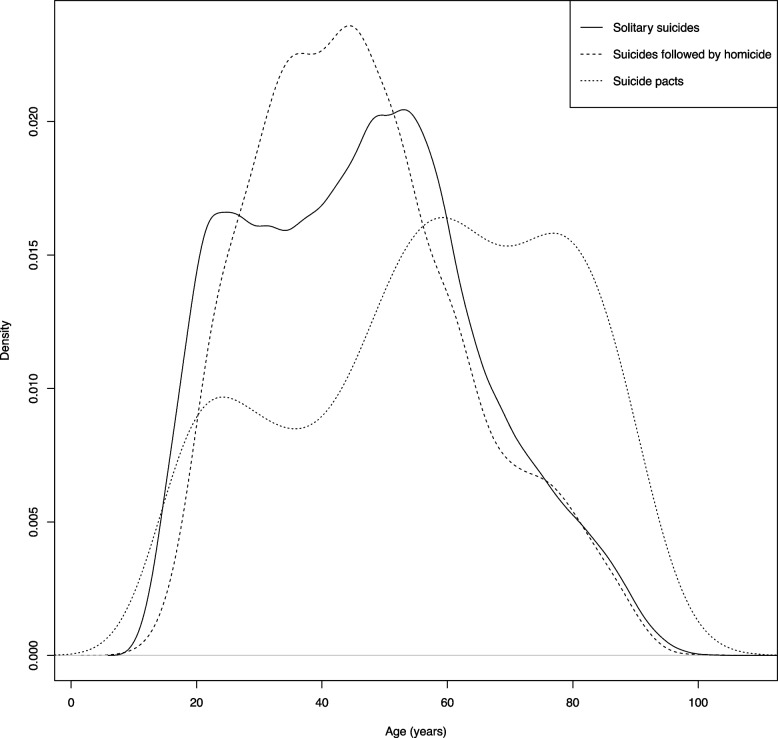
Age distribution of solitary suicides, suicides following homicide, and suicide pacts

**Fig. 2:**
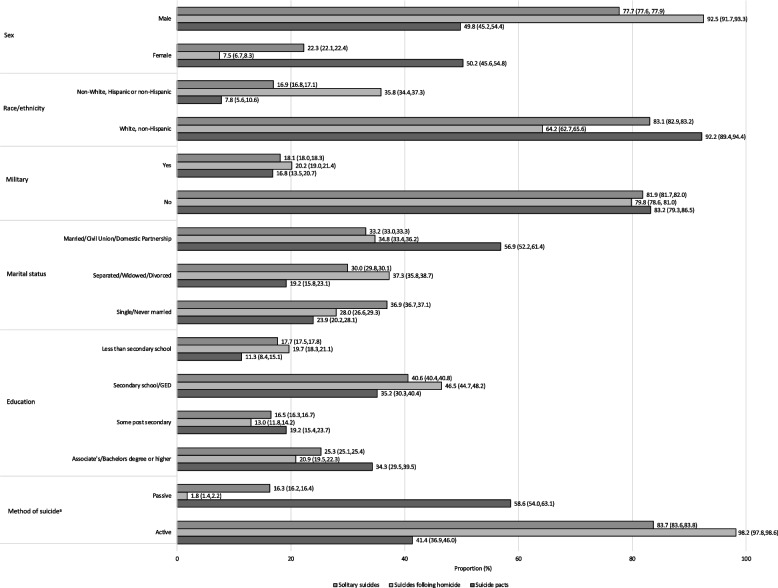
Demographics of decedents of solitary suicides, suicides following homicide, and suicide pacts *Note.* Confidence interval presented in brackets ^a^Excludes incidents where method of suicide was unknown

Suicide pact decedents were on average older than decedents of solitary suicides and suicides following homicide (mean age 57.1 years (SD = 21.7), 46.3 years (SD = 18.3), and 46.3 years (SD = 16.6), respectively). The age distribution appears to be bimodal for solitary suicides (peaking around 20 years and then again between 50 and 60 years), unimodal for suicides following homicide (peaking around 45 years; the largest proportion being between 30 and 50 years), and trimodal for suicide pacts (peaking around 20, 60 and 80 years; with the largest proportion being between 60 and 80 years). See Fig. 1 for the age distribution of each suicide incident type. For all pairwise comparisons of the three suicide incident types (i.e., solitary suicides vs. suicides following homicide, solitary suicides vs. suicide pacts, and suicide pacts vs. suicides following homicide), a significant association (*p* < 0.001) was found for sex, race/ethnicity, marital status, and education (Fig. 2, and Table A2 in the Appendix). When compared separately, race and ethnicity were both significantly different for all pairwise tests between suicide incident types (*p* < 0.001, *p* < 0.001, and *p* < 0.001 for solitary suicides vs. suicides following homicide, solitary suicides vs. suicide pacts, and suicide pacts vs. suicides following homicide, respectively). Suicide pacts had the highest proportion of females (50.2%) followed by solitary suicides (22.3%) and then suicides following homicide (7.5%). Suicides following homicide had a higher proportion of non-white, Hispanic or non-Hispanic (35.8%) compared to both solitary suicides (16.9%) and suicide pacts (7.8%). With respect to marital status, solitary suicides had the greatest proportion of decedents who were single or never married (36.9%), suicide pacts had the greatest proportion of decedents who were married, in a civil union, or in a domestic partnership (56.9%), and suicides following homicide had the greatest proportion of decedents who were separated, widowed, or divorced (37.3%). Further, suicide pacts had the greatest proportion of decedents with some post-secondary education (19.2%) or higher (34.3%), and suicides following homicide had the greatest proportion of decedents with a secondary school education (46.5%) or less (19.7%). With the pairwise comparisons, a significant association between the method of suicide and each suicide category (*p* < 0.001, *p* < 0.001, and *p* < 0.001 for solitary suicides vs. suicides following homicide, solitary suicides vs. suicide pacts, and suicide pacts vs. suicides following homicide, respectively) was also found. The highest proportion of incidents involving an active method of suicide were observed among suicides following homicide (98.2%), followed by solitary suicides (83.7%), and then suicide pacts (41.4%). Compared to solitary suicides, suicides following homicide had over ten-fold higher odds of involving an active method of suicide. See Table 1 for the mean difference and odds ratios for demographics and method of suicide for each pairwise comparison of suicide incident type.

**Table 1:** Mean difference and odds ratios for demographics, method of suicide, preceding circumstances, mental health status, and toxicology findings for each pairwise comparison of suicide incident type

Variable		Solitary suicides (ref) vs. suicides following homicide	Solitary suicides (ref) vs. suicide pacts	Suicides following homicide (ref) vs. suicide pacts
**Demographics and method of suicide**				
Age, MD (CI)		NA	10.81 (8.79,12.82)	10.84 (8.72,12.95)
Sex, OR (CI)^a^	Female	0.28 (0.25,0.32)	3.52 (2.91,4.25)	12.49 (10.00,15.61)
Race/Ethnicity, OR (CI)^b^	White, non-Hispanic	0.37 (0.34,0.39)	2.42 (1.71,3.52)	6.61 (4.65,9.68)
Race, OR (CI)^c^	Black or African American	3.89 (3.60,4.20)	0.27 (0.11,0.53)	0.07 (0.03,0.14)
	Asian/Pacific Islander/American Indian/Alaska Native	1.53 (1.32,1.78)	1.12 (0.67,1.78)	0.73 (0.43,1.19)
Ethnicity, OR (CI)^d^	Non-Hispanic	0.52 (0.47,0.58)	5.60 (2.38,17.34)	10.70 (4.52,33.32)
Military, OR (CI)^e^	Yes	1.14 (1.05,1.23)	NA	NA
Marital Status, OR (CI)^f^	Separated/widowed/divorced	1.19 (1.10,1.28)	0.37 (0.29,0.48)	0.31 (0.24,0.41)
	Single/never married	0.72 (0.67,0.78)	0.38 (0.30,0.48)	0.52 (0.41,0.67)
Education, OR (CI)^g^	Secondary school/GED	1.03 (0.93,1.13)	1.35 (0.93,1.99)	1.31 (0.90,1.96)
	Some post-secondary school	0.71 (0.62,0.80)	1.81 (1.20,2.76)	2.56 (1.66,3.99)
	Associate’s/bachelor’s degree or higher	0.74 (0.66,0.83)	2.11 (1.46,3.12)	2.85 (1.93,4.27)
Method of suicide, OR (CI)^h^	Active	10.77 (8.60,13.69)	0.14 (0.11,0.17)	0.01 (0.01,0.02)
**Preceding circumstances**				
Death/suicide of family/friend, OR (CI)^i^	Yes	NA	NA	NA
Financial problem(s), OR (CI)^i^	Yes	0.76 (0.67,0.85)	1.56 (1.17,2.05)	2.06 (1.50,2.78)
Homeless, eviction, or home loss, OR (CI)^i^	Yes	0.73 (0.61,0.88)	NA	NA
Interpersonal relationship problem(s), OR (CI)^i^	Yes	8.85 (8.20,9.56)	0.29 (0.21,0.38)	0.03 (0.02,0.04)
Job and/or school problem(s), OR (CI)^i^	Yes	0.46 (0.40,0.52)	0.37 (0.22,0.58)	NA
Legal problem(s), OR (CI)^i^	Yes	2.54 (2.37,2.73)	NA	0.37 (0.27,0.52)
Physical health problem(s), OR (CI)^i^	Yes	0.33 (0.29,0.37)	2.61 (2.15,3.17)	7.88 (6.28,9.88)
Any crisis in the two weeks preceding suicide, OR (CI)^i^	Yes	5.52 (5.18,5.88)	NA	0.14 (0.11,0.18)
**Mental health status**				
Mental health problem, OR (CI)^i^	Yes	0.26 (0.24,0.28)	0.45 (0.36,0.56)	1.72 (1.36,2.16)
Mood disorder, OR (CI)^i^	Yes	0.21 (0.19,0.23)	0.35 (0.27,0.45)	1.68 (1.26, 2.21)
Suicide attempt history, OR (CI)^i^	Yes	0.16 (0.14,0.19)	0.48 (0.35,0.66)	3.01 (2.07,4.31)
Suicide intent disclosed, OR (CI)^i^	Yes	0.41 (0.37,0.45)	NA	1.94 (1.49,2.51)
**Toxicology findings**				
Amphetamines, OR (CI)^j^	Present	NA	NA	NA
Blood Alcohol Concentration, OR (CI)^k^	≥ 0.08 g/dl	NA	0.44 (0.31,0.62)	0.46 (0.31,0.66)
Cocaine, OR (CI)^j^	Present	NA	NA	NA
Opiates, OR (CI)^j^	Present	0.52 (0.46,0.59)	2.54 (1.97,3.27)	4.84 (3.64,6.44)

*CI *Confidence interval, *MD* Mean difference, *NA* Not applicable (omnibus test or pairwise comparison was not statistically significant, OR not calculated), *OR* Odds ratio, *ref*  Reference category

^a^Reference category: Male

^b^Reference category: Non-White, Hispanic or non-Hispanic

^c^Reference category: White

^d^Reference category: Hispanic

^e^Reference category: No

^f^Reference category: Married/civil union/domestic partnership

^g^Reference category: Less than secondary school

^h^Reference category: Passive

^i^Reference category: No, n/a, unknown

^j^Reference category: Not Present

^k^Reference category: < 0.08 g/dl

### Preceding circumstances

**Fig. 3:**
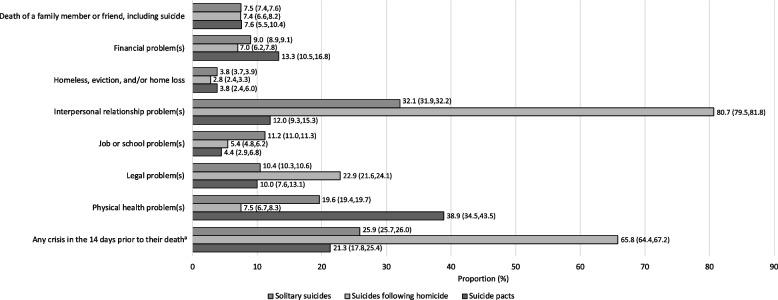
Preceding circumstances of solitary suicides, suicides following homicide, and suicide pacts *Note.* Confidence interval presented in brackets ^a^ Includes the death or suicide of a family member or friend, financial problem, homelessness, home loss or eviction, interpersonal relationship problems, job or school problems, legal problems, and/or physical health problems in the 14 days prior to their death

With the exception of experiencing the death of a family member or friend, a significant association was found for each preceding circumstance in all omnibus tests of the three suicide incident types (Fig. 3, and Table A3 in the Appendix). Suicides following homicide had a higher proportion of decedents who had had any crisis in the 14 days prior to their death (65.8%) compared with solitary suicides (25.9%; *p* < 0.001) and suicide pacts (21.3%; *p* < 0.001). Suicides following homicide had a higher proportion of decedents with interpersonal relationship problems (80.7%), compared with solitary suicides (32.1%; *p* < 0.001) and suicide pacts (12.0%; *p* < 0.001), as well as a higher proportion of decedents with legal problems (22.9%) compared with solitary suicides (10.4%; *p* < 0.001) and suicide pacts (10.0%; *p* < 0.001). Compared to solitary suicides, suicides following homicide had nearly nine-fold higher odds of having interpersonal problems and three-fold higher odds of having legal problems prior to their death (Table 1). Suicide pacts had a higher proportion of decedents with physical health problems (38.9%) compared with solitary suicides (19.6%; *p* < 0.001) and suicides following homicides (7.5%; *p* < 0.001). Suicide pacts had 2.6-fold and 7.9-fold higher odds of having physical health problems, compared to solitary suicides and suicides following homicide, respectively. See Table 1 for the odds ratios for preceding circumstances for each pairwise comparison of suicide incident type.

### Mental health status

**Fig. 4:**
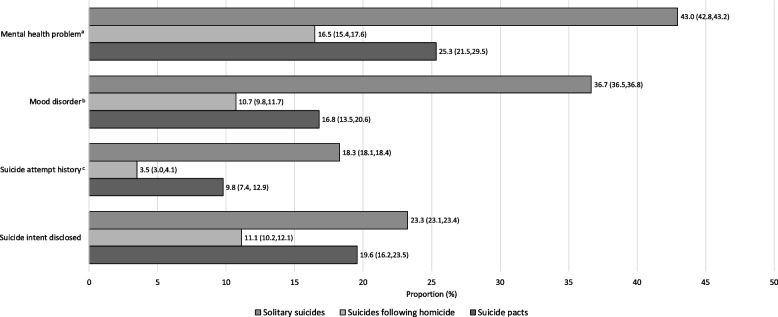
Mental health status of decedents of solitary suicides, suicides following homicide, and suicide pacts *Note.* Confidence interval presented in brackets ^a^ Any mental health problem that the victim was experiencing at the time of their death; ^b^ Bipolar disorder, depression or dysthymia; ^c^ Whether the decedent disclosed suicidal thoughts to anyone in the month preceding their death

A significant association was found between all mental health status variables in all pairwise comparisons of the three suicide incident types, with the exception of suicide intent disclosure when comparing solitary suicides and suicide pacts (Fig. 4, and Table A4 in the Appendix). Notably, the presence of mental health problems, mood disorders, and suicide attempt history were significantly different (with a *p*-value < 0.001) in all pairwise comparisons. Specifically, decedents of solitary suicides had the highest proportion of mental health problems (43.0%), mood disorders (36.7%), and suicide attempt history (18.3%), followed by suicide pacts (25.3%, 16.8%, and 9.8%, respectively), and suicides following homicide (16.5%, 10.7%, and 3.5%, respectively). Suicides following homicide and suicide pacts had 74% and 55% lower odds, respectively, of having a mental health problem and 79% and 65% lower odds, respectively, of having a mood disorder, compared to solitary suicides. See Table 1 for the odds ratios for mental health status for each pairwise comparison of suicide incident type.

### Toxicology findings

**Fig. 5:**
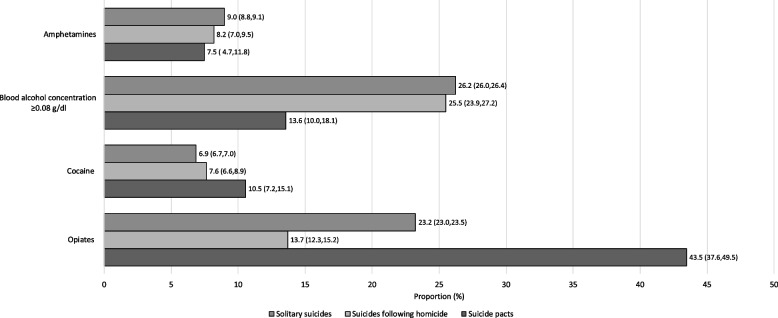
Positive toxicology findings of suicide decedents of solitary suicides, suicides following homicide, and suicide pacts *Note.* Proportions indicate the presence of the respective substances; confidence interval presented in brackets

A significant association was found for all omnibus tests of toxicology results, except for presence of amphetamines (Fig. 5, and Table A5 in the Appendix). A lower proportion of suicide pact decedents had a BAC ≥ 0.08 g/dl (13.6%) compared with decedents of solitary suicides (26.2%; *p* < 0.001) and suicides following homicides (25.5%; *p* < 0.001). Suicide pact decedents had 56% and 54% lower odds of having a BAC ≥ 0.08 g/dl, compared to solitary suicides and suicides following homicide, respectively. The presence of opiates was significantly different across all pairs (*p* < 0.001, *p* < 0.001, and *p* < 0.001 for solitary suicides vs. suicides following homicide, solitary suicides vs. suicide pacts, and suicide pacts vs. suicides following homicide, respectively), with suicide pacts having the highest proportion of decedents with opiates present (43.5%), followed by solitary suicides (23.2%) and suicides following homicide (13.7%). Compared to decedents of solitary suicides and suicides following homicide, suicide pact decedents had 2.5-fold and 4.8-fold higher odds of having taken opiates prior to their death. See Table 1 for the odds ratios for toxicology findings for each pairwise comparison of suicide incident type.

## Discussion

The current comparative descriptive analysis shows that although there are a few commonalities between solitary suicides, suicides following homicide, and suicide pacts, these three types of suicide incidents represent distinct phenomena. The numerous statistically significant differences found in the various comparisons illustrate this, and these differences have important public health implications. Distinguishing between the decedents of the different suicide incident types provides insight into points of contact where intervention and prevention can occur, which appear to be relatively distinct for each of the suicide incident types. For example, solitary suicides had the highest proportion of decedents with mental health problems and mood disorders, which suggests that mental health professionals and treatment can play an important preventive role in such incidents. In contrast, there was a comparatively lower proportion of decedents of suicides followed by homicide with physical health issues, mental health problems, past suicide attempts, and those who had disclosed their intent. This suggests that prevention efforts within the health care system may not be as effective in preventing this suicide incident type. Suicides following homicide had the highest proportion of decedents with legal and interpersonal relationship problems as well as the highest proportion of incidents involving active methods of suicide. Increased odds of firearm use, an active method of suicide, in suicides following homicides compared to solitary suicides has been previously reported [4]. Taken together, the legal system may be an area where targeted individual-level prevention efforts could prove to be beneficial for suicides followed by homicide, particularly in regards to domestic issues that escalate to the point of legal involvement [12]. At the population-level, harsher gun-restriction policies could be a beneficial prevention mechanism.

Suicide pact decedents were on average older and had the highest proportion of physical health problems, as well as opiates present in toxicology screening. This indicates that primary health care providers and health systems may play a crucial role in the prevention of suicide pacts. The high presence of opiates among suicide pact decedents (who were, on average, older than decedents of other suicide types) likely reflects their use of prescription opioids for physical ailments associated with aging such as chronic pain, cancer, etc., as opposed to use of illegal or street-sourced opioids. This underscores a potential increased opportunity for prevention through various contact points within the health care system such as oncologists, palliative care specialists, and/or pain management physicians. It is also interesting to note that despite the high proportion with physical health issues, a lower proportion, compared to solitary suicides, had a mental health problem or mood disorder. This finding could indicate that there is a need for better coordination and collaboration between health care system sectors in order to address the mental health needs of those experiencing physical health problems. There were also a lower proportion of suicide pact decedents with BAC ≥ 0.08 g/dl when compared to the other suicide incident types. This suggests a potential lower level of impulsivity more planning and a longer prevention interval. Finally, suicide pacts had a much lower proportion of decedents who used an active method of suicide compared to the other suicide incident types.

Further, understanding the demographic differences of these three types of suicide incidents will help to identify target groups for prevention efforts. The World Health Organization’s *Live Life* approach to suicide prevention suggests “early identify, assess, manage and follow up anyone affected by suicidal behaviours” as an effective evidence-based intervention to prevent suicide [13]. Characterizing decedents of solitary suicides, suicides following homicide, and suicide pacts, can ultimately inform who is most at-risk and thus, aid in the early identification of individuals likely to engage in suicidal behavior.

The results of the current study are comparable to previous studies comparing solitary suicides to suicides following homicide, as well as solitary suicides to suicide pacts. Aside from the findings of the current literature summarized above, previous studies identified firearms to be more commonly used in suicides following homicide than in solitary suicides [4, 6, 14], which is reflected in the higher proportion of active methods of suicide in suicides following homicides found here. However, it is worth highlighting that the current study is the first to compare a number of variables across all three suicide incident types (e.g., military status, education, and presence of amphetamines, cocaine, opiates, just to name a few). Further, this is the first study to compare solitary suicides, suicides following homicide, and suicide pacts simultaneously.

With that being said, the current study is not without its limitations. First, despite there being 44 participating states in 2019, not all states are proportionally represented, which also limits our ability to compare suicide types by geographic region. Second, the majority of preceding circumstances and mental health variables are binary variables in the NVDRS. As such, although we can be sure that “yes” means the variable is present, we cannot be sure that “no, not available, and unknown” means the variable is not present. NVDRS data abstractors are limited to the information included in investigative reports; reports that are incomplete, inaccurate, or unavailable may lead to underreporting or misreporting of circumstances and characteristics for some decedents or incidents. This leads to missing data, the extent of which differs by variable, which can be appreciated by the counts provided in the Appendix (Tables A2-A5). Third, as per the NVDRS data sharing agreement, some variable categories had to be collapsed in order to supress cells with fewer than ten deaths. In addition to other variables (e.g., method of suicide), this was the case for race/ethnicity. Given that the US is a diverse nation, collapsing race/ethnicity into binary categories may therefore not accurately represent the US population. In the future, when more data becomes available (particularly for suicide pacts), reporting disaggregated data might reveal inequities and disparities in subpopulations that may be obscured by reporting aggregated data. Finally, toxicology findings are only available for decedents for whom a toxicology examination was performed, and the presence of the respective substances were ascertained. Toxicology testing is not supported by Centers for Disease Control and Prevention funding and thus depends greatly on local resources. As such, some states have limited toxicology data due to the cost of testing [15]. In such states, toxicological data are most often collected only from decedents for whom this information is important for the determination of the cause of death. It is possible that when an active method of suicide is involved the cause of death is more easily identifiable than when a passive method is used; and as such, suicides that involved an active method could have lower rates of toxicological testing, compared to passive methods. In addition, there is a potential racial disparity as certain races may be more likely to have an autopsy performed (for example, the white race may be tested less often than other races [16]).

This study is intended to lay the foundation for future studies and will hopefully inspire further investigation into the similarities and differences between the three suicide incident types under investigation here. Areas of future research should explore whether the differences noted here vary by age group.

## Conclusion

Overall, based on the findings of the current investigation, we can conclude there are notable differences between solitary suicides, suicides following homicide, and suicide pacts with respect to the demographic factors, method of suicide, preceding circumstances, mental health status, and toxicology findings of decedents. This differentiation of suicide incident types is intended to provide insight into the prevention pathways that need to be exploited, some of which are likely untapped.

## Supplementary Information

**Additional file 1:** **Table A1.** NVDRS participatingstates by year, which were included in the current study. **Table A2**. Demographics andmethod of suicide, by suicide incident type. **Table A3**. Precedingcircumstances, by suicide incident type. **Table A4**.Mental health status,by suicide incident type. **Table A5**. Toxicology findings, bysuicide incident type.

## Data Availability

The data that support the findings of this study are available from the Centers for Disease Control and Prevention, National Center for Injury Prevention and Control. Restrictions apply to the availability of these data, which were used under agreement for this study.
