# Supplementary material for: A comparative analysis of solitary suicides, suicides following homicide, and suicide pacts using the National Violent Death Reporting System
Source: BMC Psychiatry. 2023 Jan 3;23:1. doi: 10.1186/s12888-022-04495-w (PMC9808963; doi:10.1186/s12888-022-04495-w)
Supplement: Supplementary file 1 — Additional file 1: Table A1. NVDRS participatingstates by year, which were included in the current study. Table A2. Demographics andmethod of suicide, by suicide incident type. Table A3. Precedingcircumstances, by suicide incident type. Table A4.Mental health status,by suicide incident type. Table A5. Toxicology findings, bysuicide incident type. [file 12888_2022_4495_MOESM1_ESM.docx]

**SUPPLEMENTARY MATERIAL**

**Table A1.** NVDRS participating states by year, which were included in the current study

| State | 2003 | 2004 | 2005 | 2006 | 2007 | 2008 | 2009 | 2010 | 2011 | 2012 | 2013 | 2014 | 2015 | 2016 | 2017 | 2018 | 2019 |
| --- | --- | --- | --- | --- | --- | --- | --- | --- | --- | --- | --- | --- | --- | --- | --- | --- | --- |
| Alabama |  |  |  |  |  |  |  |  |  |  |  |  |  |  |  | X | X |
| Alaska | X | X | X | X | X | X | X | X | X | X | X | X | X | X | X | X | X |
| Arizona |  |  |  |  |  |  |  |  |  |  |  |  | X | X | X | X | X |
| California |  |  |  |  |  |  |  |  |  |  |  |  |  |  | X^a^ | X^b^ | X^c^ |
| Colorado |  | X | X | X | X | X | X | X | X | X | X | X | X | X | X | X | X |
| Connecticut |  |  |  |  |  |  |  |  |  |  |  |  | X | X | X | X | X |
| Delaware |  |  |  |  |  |  |  |  |  |  |  |  |  |  | X | X | X |
| District of Columbia |  |  |  |  |  |  |  |  |  |  |  |  |  |  | X | X | X |
| Georgia |  | X | X | X | X | X | X | X | X | X | X | X | X | X | X | X | X |
| Hawaii |  |  |  |  |  |  |  |  |  |  |  |  | X | X |  |  | X |
| Illinois |  |  |  |  |  |  |  |  |  |  |  |  |  | X^d^ | X^d^ | X^d^ | X^d^ |
| Indiana |  |  |  |  |  |  |  |  |  |  |  |  |  | X | X | X | X |
| Iowa |  |  |  |  |  |  |  |  |  |  |  |  |  | X | X | X | X |
| Kansas |  |  |  |  |  |  |  |  |  |  |  |  | X | X | X | X | X |
| Kentucky |  |  | X | X | X | X | X | X | X | X | X | X | X | X | X | X | X |
| Louisiana |  |  |  |  |  |  |  |  |  |  |  |  |  |  |  | X | X |
| Maine |  |  |  |  |  |  |  |  |  |  |  |  | X | X | X | X | X |
| Maryland | X | X | X | X | X | X | X | X | X | X | X | X | X | X | X | X | X |
| Massachusetts | X | X | X | X | X | X | X | X | X | X | X | X | X | X | X | X | X |
| Michigan |  |  |  |  |  |  |  |  |  |  |  | X | X | X | X | X | X |
| Minnesota |  |  |  |  |  |  |  |  |  |  |  |  | X | X | X | X | X |
| Missouri |  |  |  |  |  |  |  |  |  |  |  |  |  |  |  | X | X |
| Montana |  |  |  |  |  |  |  |  |  |  |  |  |  |  |  |  | X |
| Nebraska |  |  |  |  |  |  |  |  |  |  |  |  |  |  |  | X | X |
| Nevada |  |  |  |  |  |  |  |  |  |  |  |  |  |  | X | X | X |
| New Hampshire |  |  |  |  |  |  |  |  |  |  |  |  | X | X | X | X | X |
| New Jersey | X | X | X | X | X | X | X | X | X | X | X | X | X | X | X | X | X |
| New Mexico |  |  | X | X | X | X | X | X | X | X | X | X | X | X | X | X | X |
| New York |  |  |  |  |  |  |  |  |  |  |  |  | X | X | X | X |  |
| North Carolina |  | X | X | X | X | X | X | X | X | X | X | X | X | X | X | X | X |
| North Dakota |  |  |  |  |  |  |  |  |  |  |  |  |  |  |  |  | X |
| Ohio |  |  |  |  |  |  |  |  | X | X | X | X | X | X | X | X | X |
| Oklahoma |  | X | X | X | X | X | X | X | X | X | X | X | X | X | X | X | X |
| Oregon | X | X | X | X | X | X | X | X | X | X | X | X | X | X | X | X | X |
| Pennsylvania |  |  |  |  |  |  |  |  |  |  |  |  |  | X^d^ | X^d^ | X^d^ | X^d^ |
| Puerto Rico |  |  |  |  |  |  |  |  |  |  |  |  |  |  | X | X | X |
| Rhode Island |  | X | X | X | X | X | X | X | X | X | X | X | X | X | X | X | X |
| South Carolina | X | X | X | X | X | X | X | X | X | X | X | X | X | X | X | X | X |
| Utah |  |  | X | X | X | X | X | X | X | X | X | X | X | X | X | X | X |
| Vermont |  |  |  |  |  |  |  |  |  |  |  |  | X | X | X | X | X |
| Virginia | X | X | X | X | X | X | X | X | X | X | X | X | X | X | X | X | X |
| Washington |  |  |  |  |  |  |  |  |  |  |  |  |  | X^d^ | X^d^ | X | X |
| West Virginia |  |  |  |  |  |  |  |  |  |  |  |  |  |  | X | X | X |
| Wisconsin |  | X | X | X | X | X | X | X | X | X | X | X | X | X | X | X | X |
| Wyoming |  |  |  |  |  |  |  |  |  |  |  |  |  |  |  |  | X |
| Total | 7 | 13 | 16 | 16 | 16 | 16 | 16 | 16 | 17 | 17 | 17 | 18 | 27 | 32 | 37 | 41 | 44 |

^a^ Collected data for violent deaths that occurred in 4 counties (representing 27.8% of violent deaths that occurred in California in 2017), in accordance with requirements under which the state was funded.

^b^ Collected data for violent deaths that occurred in 21 counties (representing 55.1% of violent deaths that occurred in California in 2018), in accordance with requirements under which the state was funded.

^c^ Collected data for violent deaths that occurred in 30 counties (representing 55.3% of violent deaths that occurred in California in 2019), in accordance with requirements under which the state was funded.

^d^ Collected data on >80% of violent deaths in state, in accordance with requirements under which the state was funded.

| **Variable** | | **Solitary suicides (n=262,679)** | **Suicides following homicide (n=4,352)** | **Suicide pacts (n=450)** | **Omnibus test** | | **Pairwise comparisons** | | | | | |
| --- | --- | --- | --- | --- | --- | --- | --- | --- | --- | --- | --- | --- |
|  |  |  |  |  |  |  | **Solitary suicides vs. suicides following homicide** | | **Solitary suicides vs. suicide pacts** | | **Suicide following homicide vs. suicide pacts** | |
|  |  |  |  |  | **Test statistic** | **p-value** | **Test statistic** | **p-value** | **Test statistic** | **p-value** | **Test statistic** | **p-value** |
| Age, mean (SD)^a^ | | 46.3 (18.3) | 46.3 (16.6) | 57.1 (21.7) |  |  |  |  |  |  |  |  |
|  |  |  |  |  | 78.8 | **<0.001** | 0.1 | 0.994 | 17.8 | **<0.001** | 17.0 | **<0.001** |
| Sex, n (%)^a^ | Male | 204,129 (77.7) | 4,027 (92.5) | 224 (49.8) |  |  |  |  |  |  |  |  |
|  | Female | 58,543 (22.3) | 325 (7.5) | 226 (50.2) |  |  |  |  |  |  |  |  |
|  |  |  |  |  | 753.5 | **<0.001** | 547.1 | **<0.001** | 202.1 | **<0.001** | 733.9 | **<0.001** |
| Race/ethnicity, n (%)^a^ | Non-White, Hispanic or non-Hispanic | 44,448 (16.9) | 1,558 (35.8) | 35 (7.8) |  |  |  |  |  |  |  |  |
|  | White, non-Hispanic | 218,021 (83.1) | 2,792 (64.2) | 415 (92.2) |  |  |  |  |  |  |  |  |
|  |  |  |  |  | 1098.1 | **<0.001** | 1069.1 | **<0.001** | 26.8 | **<0.001** | 144.6 | **<0.001** |
| Military, n (%)^a^ | Yes | 44,923 (18.1) | 825 (20.2) | 70 (16.8) |  |  |  |  |  |  |  |  |
|  | No | 202,598 (81.9) | 3,264 (79.8) | 347 (83.2) |  |  |  |  |  |  |  |  |
|  |  |  |  |  | 11.7 | **0.003** | 11.1 | **0.003** | 0.5 | 1.000 | 2.7 | 0.295 |
| Marital status, n (%)^a^ | Married/civil union/domestic partnership | 86,096 (33.2) | 1,490 (34.8) | 252 (56.9) |  |  |  |  |  |  |  |  |
|  | Separated/widowed/divorced | 77,799 (30.0) | 1,598 (37.3) | 85 (19.2) |  |  |  |  |  |  |  |  |
|  | Single/never married | 95,732 (36.9) | 1,198 (28.0) | 106 (23.9) |  |  |  |  |  |  |  |  |
|  |  |  |  |  | 281.8 | **<0.001** | 169.7 | **<0.001** | 112.2 | **<0.001** | 92.6 | **<0.001** |
| Education, n (%)^a^ | Less than secondary school | 36,284 (17.7) | 620 (19.7) | 39 (11.3) |  |  |  |  |  |  |  |  |
|  | Secondary school/GED | 83,459 (40.6) | 1,465 (46.5) | 121 (35.2) |  |  |  |  |  |  |  |  |
|  | Some post-secondary school | 33,907 (16.5) | 409 (13.0) | 66 (19.2) |  |  |  |  |  |  |  |  |
|  | Associate’s/bachelor’s degree or higher | 51,919 (25.3) | 658 (20.9) | 118 (34.3) |  |  |  |  |  |  |  |  |
|  |  |  |  |  | 103.8 | **<0.001** | 80.6 | **<0.001** | 22.9 | **<0.001** | 54.2 | **<0.001** |
| Method of suicide, n (%)^b^ | Passive (X60-X69) | 42,601 (16.3) | 77 (1.8) | 262 (58.6) |  |  |  |  |  |  |  |  |
|  | Active (X70-X83) | 218,796 (83.7) | 4,261 (98.2) | 185 (41.4) |  |  |  |  |  |  |  |  |
|  |  |  |  |  | 1262.4 | **<0.001** | 667.6 | **<0.001** | 583.7 | **<0.001** | 1988.8 | **<0.001** |

**Table A2.** Demographics and method of suicide, by suicide incident type

*Note.* Bolded text used to show those pairwise comparisons that are statistically significant at α = 0.05. Percentages may not add to 100% due to rounding.

^a^ Excludes incidents where variable was unknown or not available; ^b^ Excludes incidents where method of suicide was unknown.

**Table A3.** Preceding circumstances, by suicide incident type

| **Variable** | | **Solitary suicides (n=262,679)** | **Suicides following homicide (n=4,352)** | **Suicide pacts (n=450)** | **Omnibus test** | | **Pairwise comparisons** | | | | | |
| --- | --- | --- | --- | --- | --- | --- | --- | --- | --- | --- | --- | --- |
|  |  |  |  |  |  |  | **Solitary suicides vs. suicides following homicide** | | **Solitary suicides vs. suicide pacts** | | **Suicide following homicide vs. suicide pacts** | |
|  |  |  |  |  | **Test statistic** | **p-value** | **Test statistic** | **p-value** | **Test statistic** | **p-value** | **Test statistic** | **p-value** |
| Death/suicide of family/friend, n (%) | Yes | 19,616 (7.5) | 321 (7.4) | 34 (7.6) |  |  |  |  |  |  |  |  |
|  | No, n/a, unknown | 243,063 (92.5) | 4,031 (92.6) | 416 (92.4) |  |  |  |  |  |  |  |  |
|  |  |  |  |  | 0.1 | 0.972 | NA | NA | NA | NA | NA | NA |
| Financial problem(s), n (%) | Yes | 23,620 (9.0) | 303 (7.0) | 60 (13.3) |  |  |  |  |  |  |  |  |
|  | No, n/a, unknown | 239,059 (91.0) | 4,049 (93.0) | 390 (86.7) |  |  |  |  |  |  |  |  |
|  |  |  |  |  | 32.1 | **<0.001** | 21.6 | **<0.001** | 10.3 | **0.004** | 23.7 | **<0.001** |
| Homeless, eviction, or home loss, n (%) | Yes | 9,943 (3.8) | 122 (2.8) | 17 (3.8) |  |  |  |  |  |  |  |  |
|  | No, n/a, unknown | 252,736 (96.2) | 4,230 (97.2) | 433 (96.2) |  |  |  |  |  |  |  |  |
|  |  |  |  |  | 11.4 | **0.003** | 11.4 | **0.002** | <0.1 | 1.000 | 1.4 | 0.721 |
| Interpersonal relationship problem(s), n (%) | Yes | 84,197 (32.1) | 3,511 (80.7) | 54 (12.0) |  |  |  |  |  |  |  |  |
|  | No, n/a, unknown | 178,482 (67.9) | 841 (19.3) | 396 (88.0) |  |  |  |  |  |  |  |  |
|  |  |  |  |  | 4679.6 | **<0.001** | 4588.5 | **<0.001** | 83.0 | **<0.001** | 1005.8 | **<0.001** |
| Job and/or school problem(s), n (%) | Yes | 29,318 (11.2) | 237 (5.4) | 20 (4.4) |  |  |  |  |  |  |  |  |
|  | No, n/a, unknown | 233,361 (88.8) | 4,115 (94.6) | 430 (95.6) |  |  |  |  |  |  |  |  |
|  |  |  |  |  | 162.2 | **<0.001** | 142.1 | **<0.001** | 20.5 | **<0.001** | 0.8 | 1.000 |
| Legal problem(s), n (%) | Yes | 27,412 (10.4) | 995 (22.9) | 45 (10.0) |  |  |  |  |  |  |  |  |
|  | No, n/a, unknown | 235,267 (89.6) | 3,357 (77.1) | 405 (90.0) |  |  |  |  |  |  |  |  |
|  |  |  |  |  | 695.8 | **<0.001** | 695.5 | **<0.001** | 0.1 | 1.000 | 39.8 | **<0.001** |
| Physical health problem(s), n (%) | Yes | 51,467 (19.6) | 325 (7.5) | 175 (38.9) |  |  |  |  |  |  |  |  |
|  | No, n/a, unknown | 211,212 (80.4) | 4,027 (92.5) | 275 (61.1) |  |  |  |  |  |  |  |  |
|  |  |  |  |  | 511.1 | **<0.001** | 402.6 | **<0.001** | 106.0 | **<0.001** | 431.6 | **<0.001** |
| Any crisis in the two weeks preceding suicide, n (%) | Yes | 67,973 (25.9) | 2,865 (65.8) | 96 (21.3) |  |  |  |  |  |  |  |  |
|  | No, n/a, unknown | 194,706 (74.1) | 1,487 (34.2) | 354 (78.7) |  |  |  |  |  |  |  |  |
|  |  |  |  |  | 3513.4 | **<0.001** | 3506.5 | **<0.001** | 4.8 | 0.084 | 341.6 | **<0.001** |

n/a: not available; NA: not applicable

*Notes.* Bolded show those that are statistically significant at α = 0.05. Totals may not add to exactly 100% due to rounding.

| **Variable** | | **Solitary suicides (n=262,679)** | **Suicides following homicide (n=4,352)** | **Suicide pacts (n=450)** | **Omnibus test** | | **Pairwise comparisons** | | | | | |
| --- | --- | --- | --- | --- | --- | --- | --- | --- | --- | --- | --- | --- |
|  |  |  |  |  |  |  | **Solitary suicides vs. suicides following homicide** | | **Solitary suicides vs. suicide pacts** | | **Suicide following homicide vs. suicide pacts** | |
|  |  |  |  |  | **Test statistic** | **p-value** | **Test statistic** | **p-value** | **Test statistic** | **p-value** | **Test statistic** | **p-value** |
| Mental health problem, n (%) | Yes | 112,885 (43.0) | 718 (16.5) | 114 (25.3) |  |  |  |  |  |  |  |  |
|  | No, n/a, unknown | 149,794 (57.0) | 3,634 (83.5) | 336 (74.7) |  |  |  |  |  |  |  |  |
|  |  |  |  |  | 1282.4 | **<0.001** | 1227.7 | **<0.001** | 57.1 | **<0.001** | 22.2 | **<0.001** |
| Mood disorder, n (%) ^a^ | Yes | 92,794 (36.7) | 452 (10.7) | 72 (16.8) |  |  |  |  |  |  |  |  |
|  | No | 160,326 (63.3) | 3,768 (89.3) | 357 (83.2) |  |  |  |  |  |  |  |  |
|  |  |  |  |  | 1280.3 | **<0.001** | 1209.7 | **<0.001** | 72.9 | **<0.001** | 14.4 | **<0.001** |
| Suicide attempt history, n (%) | Yes | 48,032 (18.3) | 151 (3.5) | 44 (9.8) |  |  |  |  |  |  |  |  |
|  | No, n/a, unknown | 214,647 (81.7) | 4,201 (96.5) | 406 (90.2) |  |  |  |  |  |  |  |  |
|  |  |  |  |  | 656.6 | **<0.001** | 635.5 | **<0.001** | 21.8 | **<0.001** | 41.7 | **<0.001** |
| Suicide intent disclosed, n (%) | Yes | 61,093 (23.3) | 484 (11.1) | 88 (19.6) |  |  |  |  |  |  |  |  |
|  | No, n/a, unknown | 201,586 (76.7) | 3,868 (88.9) | 362 (80.4) |  |  |  |  |  |  |  |  |
|  |  |  |  |  | 358.6 | **<0.001** | 355.4 | **<0.001** | 3.5 | 0.190 | 27.6 | **<0.001** |

**Table A4.** Mental health status, by suicide incident type

n/a: not available

*Notes.* Bolded show those that are statistically significant at α = 0.05. Totals may not add to exactly 100% due to rounding.

^a^ Excludes incidents where variable was unknown or NA.

**Table A5.** Toxicology findings, by suicide incident type

| **Variable** | | **Solitary suicides (n=262,679)** | **Suicides following homicide (n=4,352)** | **Suicide pacts (n=450)** | **Omnibus test** | | **Pairwise comparisons** | | | | | |
| --- | --- | --- | --- | --- | --- | --- | --- | --- | --- | --- | --- | --- |
|  |  |  |  |  |  |  | **Solitary suicides vs. suicides following homicide** | | **Solitary suicides vs. suicide pacts** | | **Suicide following homicide vs. suicide pacts** | |
|  |  |  |  |  | **Test statistic** | **p-value** | **Test statistic** | **p-value** | **Test statistic** | **p-value** | **Test statistic** | **p-value** |
| Amphetamines, n (%) ^c^ | Present | 9,536 (9.0) | 158 (8.2) | 16 (7.5) |  |  |  |  |  |  |  |  |
|  | Not Present | 96,713 (91.0) | 1,775 (91.8) | 198 (92.5) |  |  |  |  |  |  |  |  |
|  |  |  |  |  | 2.1 | 0.355 | NA | NA | NA | NA | NA | NA |
| Blood Alcohol Concentration, n (%) ^c^ | ≥ 0.08 g/dl | 37,570 (26.2) | 677 (25.5) | 38 (13.6) |  |  |  |  |  |  |  |  |
|  | < 0.08 g/dl | 105,742 (73.8) | 1,977 (74.5) | 242 (86.4) |  |  |  |  |  |  |  |  |
|  |  |  |  |  | 23.7 | **<0.001** | 0.7 | 1.000 | 23.1 | **<0.001** | 19.6 | **<0.001** |
| Cocaine, n (%) ^c^ | Present | 7,649 (6.9) | 157 (7.6) | 25 (10.5) |  |  |  |  |  |  |  |  |
|  | Not Present | 103,894 (93.1) | 1,902 (92.4) | 212 (89.5) |  |  |  |  |  |  |  |  |
|  |  |  |  |  | 6.9 | **0.033** | 1.9 | 0.517 | 5.0 | 0.074 | 2.5 | 0.344 |
| Opiates, n (%) ^c^ | Present | 26,917 (23.2) | 280 (13.7) | 113 (43.5) |  |  |  |  |  |  |  |  |
|  | Not Present | 88,899 (76.8) | 1,766 (86.3) | 147 (56.5) |  |  |  |  |  |  |  |  |
|  |  |  |  |  | 163.9 | **<0.001** | 103.4 | **<0.001** | 59.4 | **<0.001** | 144.7 | **<0.001** |

NA: not applicable

*Notes.* Bolded show those that are statistically significant at α = 0.05. Totals may not add to exactly 100% due to rounding.

^c^ Excludes incidents where toxicology screening was not completed for the respective substance.
